# Stability of the pneumococcal population structure in Massachusetts as PCV13 was introduced

**DOI:** 10.1186/s12879-015-0797-z

**Published:** 2015-02-18

**Authors:** Qiuzhi Chang, Abbie E Stevenson, Nicholas J Croucher, Grace M Lee, Stephen I Pelton, Marc Lipsitch, Jonathan A Finkelstein, William P Hanage

**Affiliations:** Department of Epidemiology, Harvard School of Public Health, 677 Huntington Avenue, Boston, MA USA; Department of Infectious Disease Epidemiology, Imperial College London, London, W2 1PG UK; Center for Child Health Care Studies, Department of Population Medicine, Harvard Pilgrim Health Care Institute and Harvard Medical School, Boston, MA USA; Division of Infectious Diseases, Boston Children’s Hospital, Boston, MA USA; Maxwell Finland Laboratories, Boston University School of Medicine, Boston, MA USA; Division of General Pediatrics, Boston Children’s Hospital, Boston, MA USA

**Keywords:** Pneumococcal conjugate vaccine, Streptococcus pneumoniae, Colonization, Molecular epidemiology, MLST

## Abstract

**Background:**

The success of 7-valent pneumococcal conjugate vaccination (PCV-7) introduced to the US childhood immunization schedule in 2000 was partially offset by increases in invasive pneumococcal disease (IPD) and pneumococcal carriage due to non-vaccine serotypes, in particular 19A, in the years that followed. A 13-valent conjugate vaccine (PCV-13) was introduced in 2010. As part of an ongoing study of the response of the Massachusetts pneumococcal population to conjugate vaccination, we report the findings from the samples collected in 2011, as PCV-13 was introduced.

**Methods:**

We used multilocus sequence typing (MLST) to analyze 367 pneumococcal isolates carried by Massachusetts children (aged 3 months-7 years) collected during the winter of 2010–11 and used eBURST software to compare the pneumococcal population structure with that found in previous years.

**Results:**

One hundred and four distinct sequence types (STs) were found, including 24 that had not been previously recorded. Comparison with a similar sample collected in 2009 revealed no significant overall difference in the ST composition (p = 0.39, classification index). However, we describe clonal dynamics within the important replacement serotypes 19A, 15B/C, and 6C, and clonal expansion of ST 433 and ST 432, which are respectively serotype 22F and 21 clones.

**Conclusions:**

While little overall change in serotypes or STs was evident, multiple changes in the frequency of individual STs and or serotypes may plausibly be ascribed to the introduction of PCV-13. This 2011 sample documents the initial impact of PCV-13 and will be important for comparison with future studies of the evolution of the pneumococcal population in Massachusetts.

**Electronic supplementary material:**

The online version of this article (doi:10.1186/s12879-015-0797-z) contains supplementary material, which is available to authorized users.

## Background

The 7-valent pneumococcal conjugate vaccine (PCV-7), which targeted seven pneumococcal serotypes (4, 6B, 9V, 14, 18C, 19F, and 23F), was introduced in 2000 to the US childhood immunization schedule. Following the introduction, substantial reductions in rates of invasive pneumococcal disease (IPD) and asymptomatic carriage caused by vaccine serotypes were observed [1,2]. However, increased carriage of pneumococci of non-vaccine types (NVTs) was observed in the years that followed PCV-7 introduction. This “serotype replacement” has been suggested to be a result of vaccine selective pressure offering a relative advantage to non-vaccine types [3]. Serotype 19A strains became a particular cause for concern, being frequently isolated from IPD and often resistant to multiple classes of antibiotics [4]. In 2010, a 13-valent pneumococcal conjugate vaccine (PCV-13) was introduced for routine immunization of infants and young children. In addition to the serotypes included in PCV-7, PCV-13 targets 19A strains, as well as serotypes 1, 3, 5, 6A and 7F. It is hoped that PCV-13 will reduce morbidity due to pneumococcal serotypes not included in PCV-7 and, in particular, protect against IPD caused by serotypes common in the developing world [5].

Previous work has probed the processes leading to the emergence of NVTs during the PCV-7 era [6-8]. These have in large part been due to the outgrowth of previously uncommon NVT lineages. In some cases, successful strains that were originally of vaccine serotype acquired a NVT capsule via homologous recombination at the capsular locus, and these NVT variants have become more frequent during the PCV-7 era [9-11]. The replacement of the capsular locus through homologous recombination is termed ‘serotype switching’. Much uncertainty remains about the population level impact of serotype switching and replacement on carriage and invasive pneumococcal disease, in particular following the introduction of PCV-13. To fully characterize the pneumococcal population and to identify serotype switching, multilocus sequence typing (MLST) can further distinguish the background genotype of a particular isolate [12]. MLST characterizes strains based on the combination of alleles at seven housekeeping loci, which together determine the sequence type (ST). STs can then be used to characterize clones and their properties [13]. Serotype switching can be readily identified by the presence of isolates that share the same ST, but differ in their capsule. MLST has been used previously to document the origins of clones and to show that several important 19A strains were derived from vaccine serotype ancestors via this mechanism [7,8].

We have previously collected community samples of pneumococci from young children in Massachusetts in 2001, 2004, 2007, and 2009 and used MLST to determine the population structure and detailed molecular epidemiology [6-8]. Here, we report the results of MLST analyses of the 2011 samples, showing the further evolution of the pneumococcal population compared to samples from the same communities in 2009 [8]. While the overall carriage prevalence did not change substantially from 28.8% in 2009 to 31.5% in 2011, changes have already been observed in the prevalence of vaccine serotype carriage. Specifically, a substantial decline in 19A and the emergence of the interconverting 15B/C as the most common serotype have been observed in the early stages following the introduction of PCV-13 in Massachusetts [14]. These results will allow us to compare future trends and changes in the pneumococcal population with continued PCV13 use.

## Methods

### Data collection

Methods for sample collection and processing have been previously described in detail [6-8]. Briefly, carried pneumococcal strains were collected by nasopharyngeal swabs between October 2010 and April 2011 from children 3 months to 7 years of age attending pediatric and family medicine practices for well-child or sick visits in the same 8 Massachusetts communities as in our previous work [1,15,16], but with one additional site (Boston Medical Center) to include subjects residing in urban Boston. Parental consent was obtained by study staff and nasopharyngeal swabs were obtained by trained nurses. All study procedures were approved by the Harvard Pilgrim Health Care institutional review board. Samples were processed for *S. pneumoniae* growth, antimicrobial susceptibility using E test, and serotype using the Quellung reaction as previously described [16]. Standard Clinical and Laboratory Standards Institute (CLSI) susceptibility cutoffs were used to classify organisms as susceptible, intermediate, or resistant to the following antibiotics: amoxicillin, benzylpenicillin, ceftriaxone, clindamycin, erythromycin, levofloxacin, rifampin, trimethoprim-sulfamethoxazole, and vancomycin [17]. Strains were maintained as glycerol stocks at −80°C and DNA was purified using DNeasy tissue kits (Qiagen, Valencia, CA). To maintain consistency with our previous studies, we consider 15B and 15C to be a single, rapidly interconverting serotype, 15B/C. The results from the 2010–2011 dataset were compared with 291 carriage isolates collected from Massachusetts communities in the 2008–2009 season, which have previously been described in detail [8].

### Multilocus sequence typing (MLST)

Sequence types (STs) of isolates were determined by MLST as previously described [12]. Sequences of each of the seven gene fragments used in the pneumococcal MLST scheme were obtained from both DNA strands with an ABI 3700 or ABI 3730xl DNA analyzer. The sequences were aligned and trimmed to defined start and end positions using MEGA version 4 [18]. Allele and ST assignments were made using the MLST website (spneumoniae.mlst.net). All alleles not already present in the pneumococcal MLST database were verified by re-sequencing the gene fragment on both strands. For STs found among isolates of more than one serotype the MLST loci were re-sequenced and serotypes were confirmed by Quellung reaction. Cases of serotype switching were identified as isolates of the same ST (or with allelic profiles differing at one of the seven MLST loci), but which have different serotypes.

### Analysis

Differences in the ST composition of the 2009 and 2011 populations were estimated using the classification index, with significance assessed using a permutation method [6,19]. The diversity of the samples was estimated using Simpson's index of diversity D [20], defined here as $D=\left(1-{\displaystyle {\sum}_{i=1}^m{x}_i^2}\right)\left(\frac{N}{N-1}\right)$ where *x* is the fraction of the sample with sequence type *i*, *m* is the total number of sequence types, and *N* is the sample size. Variance and 95% confidence intervals were calculated as previously described [21].

eBURST software was used to visually compare the population structures in 2009 and 2011 [22]. This program groups related STs sharing 6 of the 7 alleles into clonal complexes (CCs), identifies the putative ancestor of each CC, and outputs a graphical representation of these relationships. This enabled us to assess the frequency of CCs of related strains in the 2011 sample and compare them with the sample collected in 2009 [8]. Fisher’s exact test was used to test for the significance of changes in frequency of individual STs compared to all other STs within each serotype. Analyses were performed using SAS software version 9.3 (SAS Institute, Cary NC) and R statistical package.

## Results and discussion

### ST diversity

In total, 367 *S. pneumoniae* carriage isolates were available for typing. We found 104 distinct STs, 24 of which were new to the database and included several previously undocumented alleles (1 *aroe*, 4 *gdh*, 1 *spi*, 2 *xpt*, and 3 *ddl*). Those STs present 4 or more times in the sample, along with the serotypes associated with them, are summarized in Table 1. Taken together, these 26 STs make up 69% of the sample. The full results of all isolates are presented in Additional file 1. The diversity (D) of STs in the 2011 sample was 0.98 (95% CI: 0.97-0.99) This is virtually identical to that of the 2009 sample in which D was 0.98 (95% CI: 0.98-0.98), indicating no significant change in ST diversity following the introduction of PCV-13 such as might be expected from the loss of STs associated with vaccine serotype as observed following PCV-7 introduction [7,8,23]. This is consistent with the previous hypothesis that the carried pneumococcal population has reached a new equilibrium after PCV-7 use [23].

**Table 1:** **Serotypes associated with sequence types (ST) found 4 or more times among 367** ***S. pneumoniae*** **carriage isolates in children 3 months - 7 years from Massachusetts communities in 2010–2011**

**ST**	**N (%)**	**Serotypes (N)**
**199**	35 (9.5)	15B/C (33), 19A (2)
**62**	21 (5.7)	11A (21)
**320**	19 (5.2)	19A (19)
**433**	16 (4.4)	22F (16)
**63**	14 (3.8)	15A (10), 15B/C (3), 19A (1)
**558**	14 (3.8)	35B (13), 29 (1)
**338**	13 (3.5)	23A (13)
**432**	12 (3.3)	21 (12)
**180**	10 (2.7)	3 (10)
**498**	10 (2.7)	35F (10)
**695**	8 (2.2)	19A (8)
**3280**	8 (2.2)	15B/C (8)
**439**	7 (1.9)	23B (7)
**547**	7 (1.9)	34 (7)
**816**	7 (1.9)	10A (7)
**1390**	7 (1.9)	6C (7)
**138**	6 (1.6)	6C (6)
**659**	6 (1.6)	16F (6)
**162**	5 (1.4)	15B/C (5)
**7490**	5 (1.4)	21 (5)
**36**	4 (1.1)	23B (4)
**191**	4 (1.1)	7F (4)
**632**	4 (1.1)	9N (4)
**1262**	4 (1.1)	15B/C (4)
**1876**	4 (1.1)	6C (3), 6A (1)
**1924**	4 (1.1)	17F (4)

We examined whether the 2009 and 2011 samples were significantly different in their ST composition with the classification index [19]. This test calculates a statistic for the similarity between the ST content of two populations, and then randomly permutes the pooled populations into two samples of the same size to estimate how the test statistic is distributed under the null hypothesis of the two samples being from the same population. We found no significant evidence of difference between the 2009 and 2011 samples (p = 0.39 from 1000 permutations). We also applied Fisher’s exact test to assess difference in ST composition within each serotype and found no significant difference between the 2009 and the 2011 samples.

**Figure 1:**
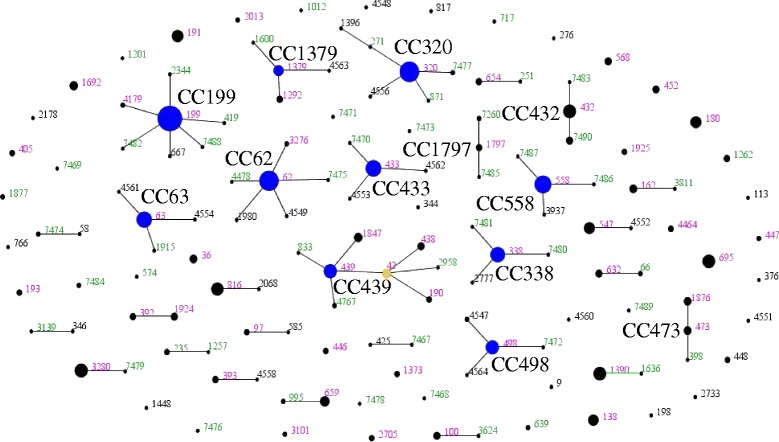
**Comparative eBURST diagram showing changes between the 2009 and 2011** ***S. pneumoniae*** **carriage isolates in children 3 months - 7 years from Massachusetts communities.** Each ST is represented by a point, the size of which is determined by the number of isolates with that ST in the combined dataset. STs differing at a single MLST locus are shown linked by a straight line. A clonal complex (CC) is a group of STs sharing 6 of 7 alleles with at least one other member of the group. STs which are the putative founders of their CC are shown in blue, with those which are thought to have given rise to subgroups shown in yellow. Those STs which cannot be linked to any other in the sample are termed singletons and appear as unlinked points. For more information see http://spneumoniae.mlst.net/eburst/. The ST numbers are colored according to whether they were found only in 2009 (black), only in 2011 (green), or in both (pink).

To allow for a broader comparison of the latest results with those obtained in 2009, we also generated a comparative eBURST population snapshot, which grouped STs into 28 clonal complexes (Figure 1). eBURST groups related STs, represented as circles, into CCs and identifies the probable ancestor (shown in blue) of each CC as the ST with the largest number of minor differences. STs shown in green were only found in the 2011 dataset. STs in black were only found in the 2009 dataset. Those in pink were found in both. As shown, all but one of the CCs were identified in both samples, indicating that that most common clones persisted in Massachusetts over the examined time span. On a broader level, 13 of the 15 CCs identified using genomic data from samples between 2001 and 2007 [10] were identified in 2011; those that were absent were the group of serotype 6A isolates of CC376, and the unusual set of non-typable isolates highly divergent from the rest of the population.

### Changes within individual serotypes

**Figure 2:**
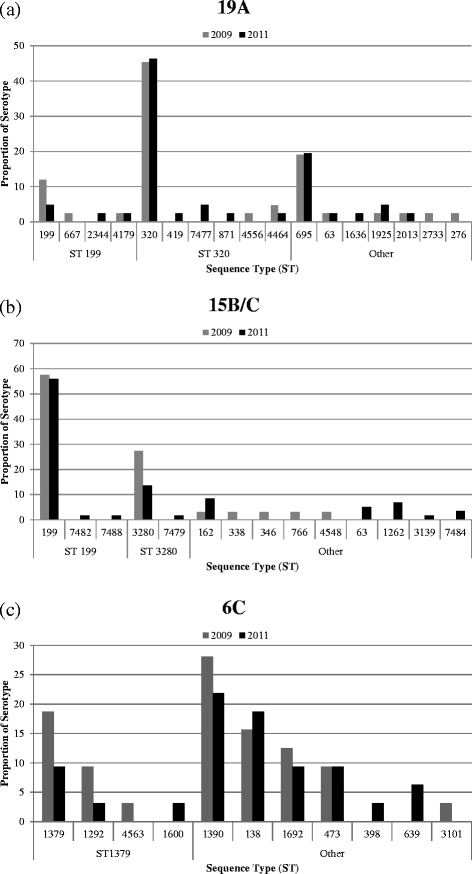
**Changes between the 2009 and 2011 samples within individual serotypes (a) 19A (b) 15B/C and (c) 6C among** ***S. pneumoniae*** **carriage isolates in children 3 months - 7 years from selected Massachusetts communities.** Results for 2009 are shown in gray and 2011 in black. STs are shown as the proportion of isolates within each serotype, arranged by clonal complex according to eBURST analysis. The ancestral ST of each clonal complex in eBURST analysis is shown below the panel. Other indicates STs for which ancestry could not be determined by eBURST.

Three serotypes are responsible for nearly half of the 367 *S. pneumoniae* carriage isolates that were collected in 2011: 19A, 15B/C, and 6C. To show changes in the clonal composition of the most common serotypes in the 2011 sample, histograms comparing the ST composition of serotypes 19A, 15B/C, and 6C in the 2009 and 2011 samples are shown in Figure 2. Using Fisher’s exact test, no statistically significant differences were observed in the ST populations within serotype 19A, 15B/C, and 6C.

Serotype 19A is of considerable concern as an important replacement serotype in carriage and invasive disease. It makes up 11% of the 2011 sample (41/367) compared to 14% of the 2009 sample (42/291). In addition, serotype 19A is represented by three predominant STs: two ST 199 (4.9% of serotype 19A), nineteen ST 320 (46.3% of serotype 19A), and eight ST 695 (19.5% of serotype 19A). Similar to the changes between 2007 and 2009, a decrease in ST 199 in 19A was observed in parallel with slight increases in ST 320 and ST 695 as shown in Figure 2(a). As previously observed and in contrast with ST 199, ST 695 has remained at a relatively constant prevalence. We also observed that ST 320 remains the most common ST for serotype 19A, making up almost half of the 19A population. ST 320 was found to be associated with high-level benzylpenicillin resistance (all isolates had minimum inhibitory concentrations ≥3 μg/mL), high-level erythromycin resistance (all but two isolates with minimum inhibitory concentrations >256 μg/mL) and high-level ceftriaxone resistance (all isolates with minimum inhibitory concentrations ≥2 μg/mL). The continued success of ST 320 may be determined by its high level of resistance to multiple antibiotic classes, giving it an advantage over ST 199. Clonal replacement among 19A pneumococcal population observed following PCV-7 has been discussed previously [8]. Serotype 19A will become increasingly important for surveillance of pneumococcal carriage and invasive disease given that serotype 19A is included in PCV-13.

Serotype 15B/C represents 16% of the 2011 sample (59/367), compared to 11% of the 2009 sample (33/291). The increase in serotype 15B/C isolates represents the combination of the stable prevalence of the 15B/C ST 199 isolates and the emergence of multiple unrelated clones including ST 63, ST 1262, ST 3139, and ST 7484, as shown in Figure 2(b). The most common ST in 15B/C strains was ST 199 (55.9% of serotype 15B/C), as has been the case since the start of sampling in 2001. In contrast, there was a decrease in the prevalence of ST 3280 (13.6% of serotype 15B/C). Strains of this ST were originally detected in a single community in 2007 exhibiting an intermediate level resistance to penicillin [10] but after rising to just over a quarter of serotype 15B/C isolates in 2009, they do not appear to achieve more widespread success. In addition, among other STs identified as 15B/C, we observed three ST 63 isolates (5.1% of serotype 15B/C) that were 15B/C, which suggests serotype switching from previous 15A strains. This is of interest as the Pneumococcal Molecular Epidemiology Network (PMEN) has recognized this as the Sweden^15A^ST63 multidrug resistant clone [24,25].

Like the other serotypes, the 6C population is made up of multiple unrelated STs. It makes up 9% of the 2011 sample (32/367), compared to 11% of the 2009 sample (32/291). As shown in Figure 2(c), we observed a decrease in ST 1379 (9.4% of serotype 6C) and related ST 1292 (3.1% of serotype 6C), as well as ST 1390 (21.9% of serotype 6C) and ST 1692 (9.4% of serotype 6C). In 2011, ST 138 made up 18.8% of the serotype 6C population. While this is not a significant increase from the 2009 sample in which it comprised 15.6% (5/32) of serotype 6C isolates in 2009, this combination of serotype and ST was not found in our samples prior to 2007, when a single isolate was recorded [7]. Historically associated with a 6B capsule, ST 138 is an example of serotype switching. We anticipate that 6C carriage may decrease in coming years as a result of cross protection from the 6A component of the vaccine [26].

### Other serotypes

The largest change in frequency between 2009 and 2011 was found for ST 180, a serotype 3 clone. Although non-significant (p = 0.076, two-tailed Fisher’s Exact test), the proportion of samples with this ST and serotype was 10/367 (2.72%) as shown in Table 1, an increase from 2/291 (0.69%) in 2009 [8]. This may be related to the low immunoglobulin G response for serotype 3, in comparison to the other additional 5 serotypes included in PCV-13. Immunogenicity studies of PCV-13 in the United States have shown that following vaccination at ages 2, 4, and 6 months, 73.3% of subjects had antibody titers against serotype 3 exceeding 0.35 μg/mL, a reference concentration for assessment of vaccine efficacy against IPD defined by the WHO [27,28]. Additional surveillance will be needed to determine whether this increase in serotype 3 continues with increased uptake of PCV-13.

In addition to ST 180, we also observed a striking increase in ST 432 and ST 433, which are associated with the non-vaccine serotypes 21 and 22F, respectively. Although statistically non-significant, ST 433 increased from 8/291 (2.75%) to 16/367 (4.36%) while ST 432 increased from 4/291 (1.37%) to 12/367 (3.27%). This is likely a result of clonal expansion following vaccination and the early stages of serotype replacement given that serotypes 21 and 22F are not included in PCV-13. Serotype 22F isolates of ST 433 have become increasingly prevalent as a cause of invasive pneumococcal disease in Japan following implementation of PCV-7 [29], and previous work using data collected in Massachusetts has suggested this serotype has a relatively high potential to cause invasive disease (on par with 19A) [30]. It is possible that we may see some non-PCV13 serotypes increase and others may emerge due to the selection pressure, although it is difficult to predict the extent of subsequent carriage and invasive disease replacement by such serotypes.

As in previous samples [6-8], we document numerous 11A isolates of ST 62. While this lineage is plainly successful, it has low invasive capacity and is not as yet associated with antibiotic resistance [30]. Future surveillance will be important to note if either of these factors change with continued high prevalence of this strain.

One isolate was found to be non-typable. The MLST of this isolate was closely related to ST 15, which was associated with serotype 14 prior to vaccination [31]. While it is not clear from the present data whether the capsule has been fully lost or down-regulated [32], a serotype 14 capsule would be targeted by the vaccine and thus loss of capsule may be an adaptation.

## Conclusions

We have previously used MLST to characterize samples of carried pneumococci from MA children collected in 2001, 2004, 2007, and 2009. The overall carriage prevalence did not change substantially from 28.8% in 2009 to 31.5% in 2011 [14]. In this paper, we report the results of the MLST analysis of the most recent sample collected in 2011. The 2011 sample was not found to be significantly different in terms of diversity as measured by Simpson’s *D* or in terms of overall ST composition (tested using the Classification Index [19]) from the 2009 sample. This is consistent with previous findings that serotype distribution has reached a new equilibrium in the presence of PCV-7 where the diversity of population structure of carried serotypes was similar to that observed in the absence of vaccination [8,23]. While it could be considered surprising that there was no change despite the introduction of PCV-13 in April 2010, it should be noted that the sample was collected as the vaccine was being introduced. Furthermore, while high levels of coverage in young infants were achieved over a short period of time, there would have been lower levels of coverage achieved for those over one year of age in our sample. Thus, we would not yet anticipate the full benefit of the direct protection and herd effect from PCV-13 vaccination due to its slow uptake, particularly among older children [14]. Subsequent sampling will be needed to study the further evolution of the pneumococcal population, to document clonal changes within serotypes and the emergence of additional non-vaccine serotypes with the increased uptake of PCV-13.

## Additional file

Additional file 1:
**Results of MLST analysis of 367 pneumococcal carriage isolates.**
